# Interstitial lung disease associated with systemic sclerosis (SSc-ILD)

**DOI:** 10.1186/s12931-019-0980-7

**Published:** 2019-01-18

**Authors:** Vincent Cottin, Kevin K. Brown

**Affiliations:** 1 National Reference Center for Rare Pulmonary Diseases, Louis Pradel Hospital, Claude Bernard University Lyon 1, 28 Avenue du Doyen Lepine, 69677 Lyon Cedex, Lyon, France; 2 National Jewish Health, 1400 Jackson Street, Denver, CO 80206 USA

## Abstract

**Background:**

Systemic sclerosis (SSc) is a rare connective tissue disease with a heterogeneous clinical course. Interstitial lung disease (ILD) is a common manifestation of SSc and a leading cause of death.

**Main body:**

All patients newly diagnosed with SSc should receive a comprehensive clinical evaluation, including assessment of respiratory symptoms, a high-resolution computed tomography (HRCT) scan of the chest, and pulmonary function tests. ILD can develop in any patient with SSc, including those with pulmonary hypertension, but the risk is increased in those with diffuse (rather than limited) cutaneous SSc, those with anti-Scl-70/anti-topoisomerase I antibody, and in the absence of anti-centromere antibody. While it can occur at any time, the risk of developing ILD is greatest early in the course of SSc, so patients should be monitored closely in the first few years after diagnosis. An increased extent of lung fibrosis on HRCT and a low forced vital capacity (FVC) are predictors of early mortality. While not all patients will require treatment, current approaches to the treatment of progressive SSc-ILD focus on immunosuppressant therapies, including cyclophosphamide and mycophenolate mofetil. In patients with severe and/or rapidly progressive disease, both haematopoietic stem cell transplantation (HSCT) and lung transplantation have been successfully used. A number of medications, including the two drugs approved for the treatment of idiopathic pulmonary fibrosis (IPF), are under active investigation as potential new therapies for SSc-ILD.

**Conclusions:**

Physicians managing patients with SSc should maintain a high level of suspicion and regularly monitor for ILD, particularly in the first few years after diagnosis.

## Background

**Fig. 1:**
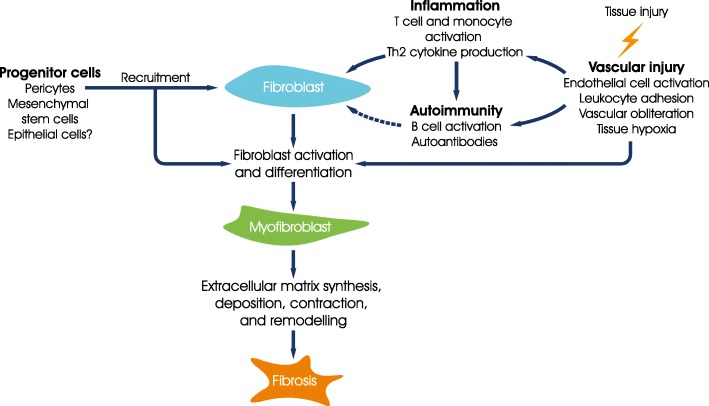
The pathogenesis of SSc (Adapted from [2]). SSc is initiated by microvascular injury, inducing inflammation, an autoimmune response, and fibroblast activation and differentiation. Activated myofibroblasts perform a series of functions, culminating in excess deposition of extracellular matrix and the development of fibrosis. Republished with permission of The Journal of Clinical Investigation, from Systemic sclerosis: a prototypic multisystem fibrotic disorder, Varga J and Abraham D, Volume No. 117, Edition No. 3, 2007; permission conveyed through Copyright Clearance Center, Inc.

Systemic sclerosis (SSc) is a rare connective tissue disease that is believed to be triggered, in genetically susceptible individuals, by environmental events. SSc is characterised by immune dysfunction, vasculopathy, cellular inflammation and fibrosis of the skin and multiple internal organs [1, 2] (Fig. 1). Patients with SSc can be classified by the extent of skin involvement: in patients with limited cutaneous SSc (lcSSc), the affected skin is restricted to the hands, forearms, feet, and face, while in patients with diffuse cutaneous SSc (dcSSc), the affected skin extends proximal to the elbows, and may involve the trunk [3].

The lung is frequently involved in SSc, with interstitial lung disease (ILD) a common manifestation [4, 5]. Indeed, ILD is included in the American College of Rheumatology (ACR)/ European League Against Rheumatism Collaborative Initiative (EULAR) joint classification criteria to identify SSc in individuals who do not have skin thickening of the fingers extending proximal to the metacarpophalangeal joints [6]. ILD associated with SSc (SSc-ILD) is usually detected during the evaluation of a patient suspected or known to have SSc, but may be the initial presentation of the disease in some patients [7].

In this article, we provide an overview of the identification, assessment, clinical course and management of SSc-ILD, including therapies under investigation.

### Diagnosis and assessment of SSc-ILD

In 2013, ACR and EULAR published new criteria for the classification of SSc [6]. The system was tested in patients with SSc and control patients with diseases similar to SSc, and validated with a group of SSc experts. The new criteria were shown to have a sensitivity of 91% and a specificity of 92% for detecting SSc. Skin thickening of the fingers extending proximal to the metacarpophalangeal joints is recognized as sufficient for a patient to be diagnosed as having SSc. If this is not present, seven other variably weighted clinical features are considered: skin thickening of the fingers, finger tip lesions (digital tip ulcers or pitting scars), telangiectasia, abnormal nailfold capillaroscopy, pulmonary arterial hypertension and/or ILD, Raynaud’s phenomenon, and SSc-related autoantibodies (anticentromere, anti-topoisomerase I, anti-RNA polymerase III).

Risk factors for the development or progression of ILD in patients with SSc include the presence of dcSSc [8], African–American ethnicity [9], older age at disease onset [8], shorter disease duration [10], and the presence of anti-Scl-70/anti-topoisomerase I antibody and/or absence of anticentromere antibody [8]. However, none of these risk factors is absolute. It is important that physicians are aware that ILD may develop in patients with limited cutaneous SSc as well as in patients with diffuse skin disease. The identification of SSc-ILD requires a high level of suspicion as not all patients will have respiratory symptoms [11]. All patients diagnosed should receive a comprehensive clinical assessment, including assessment of respiratory symptoms, chest imaging with a high resolution computed tomography (HRCT) scan, and pulmonary function tests (PFTs), to ensure early identification of ILD and provide baseline measurements to compare with future assessments. The presence of SSc-ILD is defined by the identification of fibrotic features on chest HRCT or standard chest x-ray, generally most pronounced in the lung bases, and/or when crackles that sound like ‘Velcro’ being torn apart are heard on chest auscultation (when not due to another cause) [6]. The most common imaging pattern observed on HRCT is non-specific interstitial pneumonia (NSIP) [12]. Pleuroparenchymal fibroelastosis (PPFE)-like lesions on HRCT may also be seen and appear to be associated with poor prognosis [13]. The most common histopathologic pattern seen on surgical lung biopsy is NSIP [14], though surgical lung biopsy is seldom performed in SSc patients, unless the HRCT pattern is atypical, there is suspicion of a different diagnosis, or a complication such as cancer.

The risk of developing ILD is greatest early in the course of SSc, and PFTs can be useful every 4–6 months in the first 3 years after an SSc diagnosis to ensure early detection and to monitor for progression [15]. PFTs in patients with SSc-ILD generally demonstrate a restrictive pattern, with reduced forced vital capacity (FVC) and diffusion capacity of the lung for carbon monoxide (DLco) [16]. However, even in patients with clear fibrosis on HRCT, FVC may be normal [17]. As a reduced DLco may be a result of pulmonary hypertension and/or emphysema rather than, or in addition to, ILD [18, 19], it is important that DL_CO_ be interpreted within the entire clinical context. European Society of Cardiology (ESC)/European Respiratory Society (ERS) guidelines recommend that patients with SSc should be screened for PH to ensure early detection [20].

### Clinical course of SSc-ILD

**Fig. 2:**
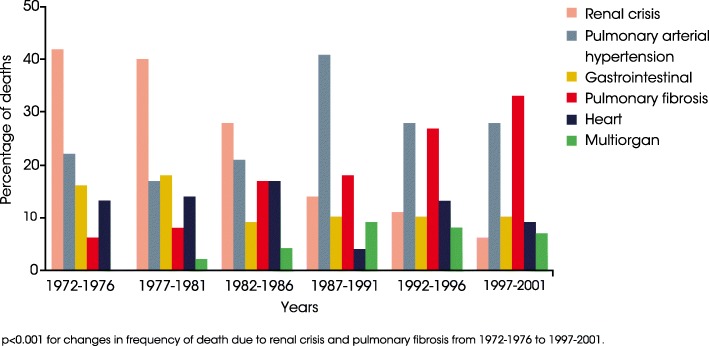
Causes of SSc-related deaths between 1972 and 2001 (Adapted from [21]). Reproduced from Ann Rheum Dis, Steen VD and Medsger TA, Volume 66, Pages 940–44, 2007, with permission from BMJ Publishing Group Ltd.

SSc-ILD is associated with early mortality. In a study of causes of death in 1508 patients with SSc from a single US centre, deaths attributed to pulmonary fibrosis increased from 6% in 1972–1976 to 33% in 1997–2001, making ILD the most frequent cause of SSc-related death (Fig. 2) [21]. Similarly, in an analysis of 5850 patients in the EULAR Scleroderma Trials and Research Group (EUSTAR) database, pulmonary fibrosis was responsible for 35% of SSc-related deaths from 2004 to 2008 [22]. An analysis of death certificates from 2719 French patients with SSc who died between 2000 and 2011 found that almost half of these deaths were due to cardiac or respiratory causes, and that the proportion of deaths related to SSc rather than other causes, increased over this period [23].

**Fig. 3:**
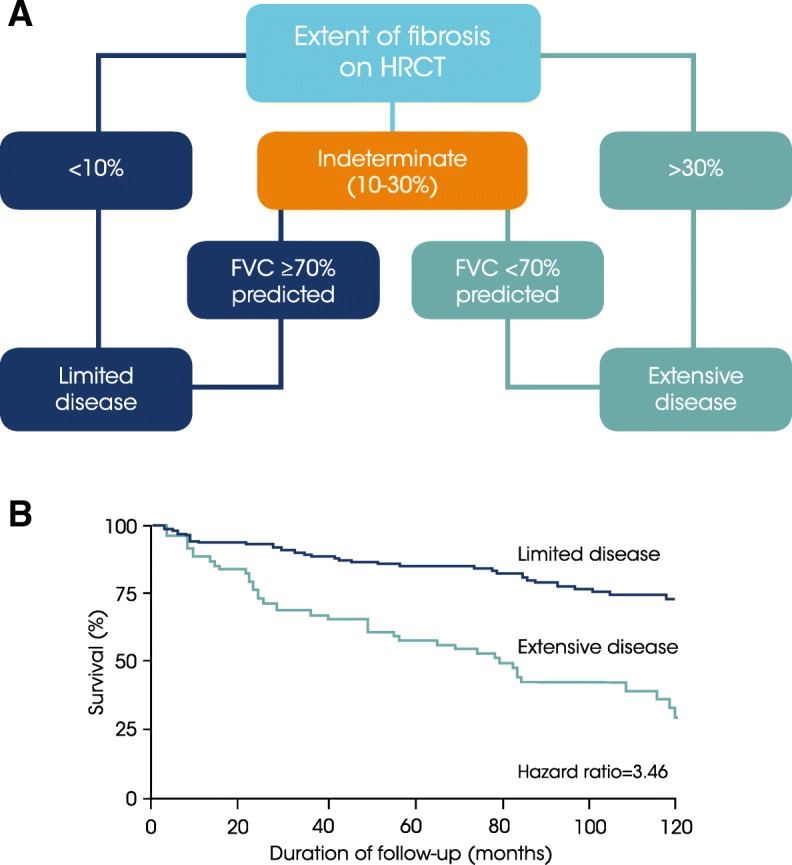
A simple staging system for prediction of survival in patients with SSc-ILD. **a** Patients with SSc may be classified as having limited disease or extensive disease based on the extent of fibrosis seen on HRCT of the lungs, plus FVC per cent predicted in patients with 10–30% fibrosis on HRCT. **b** Extensive lung disease is a significant predictor of mortality with a hazard ratio of 3.46 compared to limited disease [30]. Reprinted with permission of the American Thoracic Society. Copyright© 2018 American Thoracic Society. Goh NS et al. 2008. Interstitial lung disease in systemic sclerosis: a simple staging system. Am J Respir Crit Care Med 2008;177:1248–54. The *American Journal of Respiratory and Critical Care Medicine* is an official journal of the American Thoracic Society

SSc-ILD has a variable clinical course. Most patients will experience a slow decline in lung function, but some progress rapidly after disease onset [24–26], with progression defined by an increase in the extent of pulmonary fibrosis on HRCT or by a decline in PFTs [27–29]. Among patients in the EUSTAR cohort, 65% of patients had DLco < 80% predicted and 31% had FVC < 80% predicted 1 year after the onset of Raynaud’s phenomenon [10]. Both the extent of fibrosis on HRCT and a low FVC are independent predictors of mortality [30, 31]. A simple staging system, often referred to as the Goh criteria, which divides patients into those with extensive disease (> 30% disease extent on HRCT, or 10–30% disease extent on HRCT and FVC < 70% predicted) or limited disease (< 30% disease extent on HRCT, or 10–30% disease extend on HRCT and FVC ≥70% predicted) demonstrated that extensive disease was a powerful predictor of mortality (hazard ratio 3.46, 95% confidence interval [CI] 2.19, 5.46) (Fig. 3) [30]. In a separate study, when the prognostic significance of PFT trends at 1 year on 15-year survival were analyzed, the most accurate predictor of mortality was a relative decline in FVC of ≥10%, or a relative decline in FVC of 5–9% with a relative decline in DLco of > 15% [27]. However, neither of these prognostic indices has been prospectively studied as a guide to treatment in SSc-ILD patients. There is no evidence that the pattern of fibrosis on HRCT or histology (e.g. NSIP versus UIP) has a significant impact on disease progression or mortality in patients with SSc.

### Management of SSc-ILD

While there are no approved drugs for SSc-ILD, current approaches to treatment include routine follow-up alone (watchful waiting), or routine follow-up with active immunosuppression in patients with progressive ILD [32]. The latest treatment guidelines for SSc, issued in 2016 [33], reiterated the recommendation given in the 2009 guidelines [34] that tailored treatment with CYC should be considered, particularly in patients with progressive disease. They also included a new recommendation for consideration of autologous haematopoietic stem cell transplantation (HSCT) in selected patients with rapidly progressive SSc at risk of organ failure. Given the potential for serious adverse outcomes (including death) with HSCT, this approach requires careful evaluation of individualized risks and benefits. Importantly, the latest treatment guideline was completed prior to publication of the results of the Scleroderma Lung Study II (SLS II), which showed that treatment with mycophenolate mofetil (MMF) for 2 years had comparable efficacy with oral CYC for 1 year followed by placebo for the second year [35].

Based on the available data, treatment decisions need to be made on a case by case basis. Not all patients will need therapy; however, active treatment should be considered when there is clinically significant disease at presentation or evidence of disease progression, as measured by a decline in lung function, progression of fibrosis on HRCT, or worsening respiratory symptoms due to ILD, and it is the patient’s preference [15, 36, 37]. While treatment may not be needed initially, as disease progression may occur at any time, routine monitoring is essential [15].

#### Cyclophosphamide

**Fig. 4:**
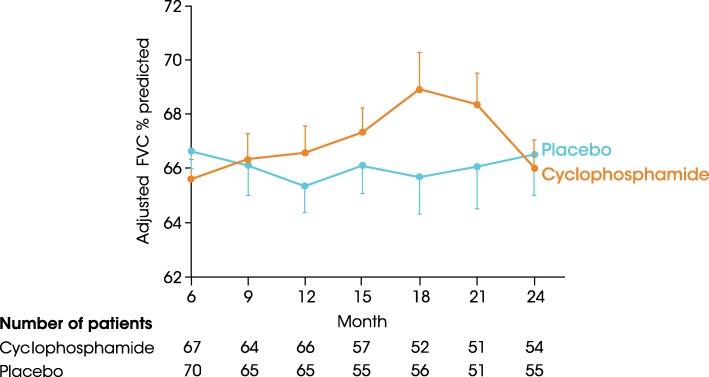
FVC % predicted in patients who received CYC or placebo for 1 year followed by an additional year of monitoring in SLS I (adapted from [39]). The vertical lines represent the standard error. FVC = forced vital capacity; CYC = cyclophosphamide. Reprinted with permission of the American Thoracic Society. Copyright© 2018 American Thoracic Society. Tashkin DP, et al. 2007. Effects of 1-year treatment with cyclophosphamide on outcomes at 2 years in scleroderma lung disease. Am J Respir Crit Care Med. 2007;176:1026–34. The *American Journal of Respiratory and Critical Care Medicine* is an official journal of the American Thoracic Society

The results of two randomised placebo-controlled trials of CYC in SSc-ILD support its use [16, 38]. In the FAST study, in which patients received low-dose prednisolone plus intravenous CYC for 6 months followed by azathioprine therapy for 6 months, or placebo for 12 months, there was a trend towards improvement in FVC with active treatment, but only 68% of patients in the active treatment group and 57% of those who received placebo completed 1 year of treatment [38]. In Scleroderma Lung Study (SLS) I, patients were randomised to receive oral CYC or placebo for 12 months, but only 54 patients (68.4%) in the CYC group and 55 (69.6%) in the placebo group completed 12 months of treatment. At month 12, there was a significant difference in favour of CYC in the primary endpoint of change from baseline in FVC % predicted (− 1.0 vs − 2.6% predicted; Fig. 4). Significant differences between CYC and placebo were also reported for changes from baseline in total lung capacity (− 0.3 vs − 2.8% predicted), skin thickness (mRSS: − 3.6 vs − 0.9 units), dyspnoea (Mahler transitional dyspnoea index: + 1.4 vs − 1.5 units) and the Health Assessment Questionnaire disability index (− 0.1 vs 0.2) at month 12 [16]. Adverse events occurred in a greater proportion of patients treated with CYC and included leucopenia (26.0% vs 0%), haematuria (12.3% vs 4.2%), neutropenia (9.6% vs 0%), pneumonia (6.8% vs 1.4%) and anaemia (2.7% vs 0%) [16]. Except for a sustained improvement in dyspnoea, the effects of CYC were not apparent 12 months after discontinuing treatment [39]. Patients with more severe disease at baseline, as measured by a greater extent of disease on HRCT or a greater extent of skin disease (higher modified Rodnan skin score [mRSS]) appeared to be more likely to respond to CYC [40]. Of note, very limited decline in FVC was observed in the placebo cohort, suggesting that the population included in this trial had relatively non-progressive ILD.

#### Mycophenolate mofetil

**Fig. 5:**
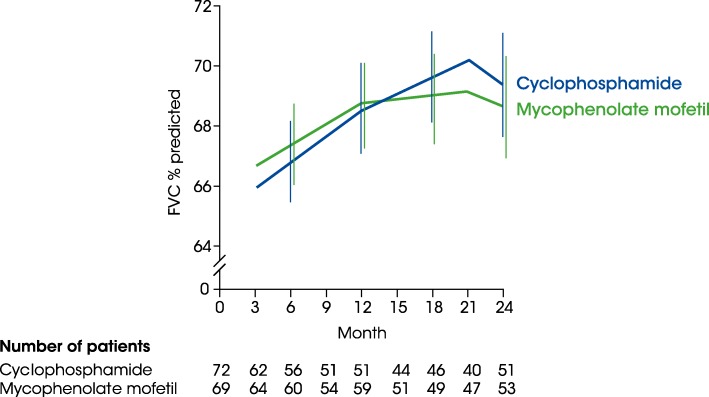
Effect of MMF for 2 years vs CYC for 1 year followed by placebo for 1 year on FVC % predicted in SLS II (adapted from [35]). The vertical lines represent the 95% CI. Reprinted from The Lancet Respir Med, Volume 4, Tashkin DP, et al. Mycophenolate mofetil versus oral cyclophosphamide in scleroderma-related interstitial lung disease (SLS II): a randomised controlled, double-blind, parallel group trial, Pages 708–19, Copyright (2016), with permission from Elsevier

MMF is commonly used in the treatment of patients with SSc-ILD [32, 41], and is more commonly used than CYC in some countries. Observational studies have suggested that MMF treatment may stabilise or even improve FVC [42]. In SLS II, there was no significant difference in the primary endpoint of change from baseline in FVC % predicted at the end of year 2 between patients who received oral MMF for two years and patients who received oral CYC for 1 year followed by placebo for 1 year (+ 2.19% vs + 2.88% predicted; difference vs CYC: -0.70, 95% CI -3.1 to 1.7; Fig. 5) [35]. Both MMF and CYC resulted in improvements from baseline to end of year 2 in Mahler transitional dyspnoea index (+ 1.77 vs + 2.16 units) and mRSS (− 4.90 vs − 5.35 units), with no significant difference between treatment groups. There were fewer treatment discontinuations due to adverse events in the MMF group than in the CYC group (35% vs 42%).

#### Haematopoietic stem cell transplantation

Randomized controlled trials comparing HSCT with CYC have shown at least stability of pulmonary physiology and an improvement in skin thickness in patients with progressive diffuse cutaneous SSc [43, 44]. In the ASTIS trial, HSCT therapy resulted in significant improvement in FVC at year 2 of follow-up, but had no impact on DLco. Significant adverse effects, including early death, occurred more frequently in the HSCT group. In the HSCT group, there were 8 treatment-related deaths (10.1%) in the first year compared with none in the CYC group. However, event-free survival was significantly greater with HSCT compared with CYC at year 1 (HR 0.52 [95% CI 0.28, 0.96]), year 2 (HR 0.35 [95% CI 0.16, 0.74] and year 4 (HR 0.34 [95% CI 0.16, 0.74]) [43]. In the ASSIST trial, HSCT and antithymocyte globulin therapy preceded by CYC and filgrastim was superior to CYC with regards to skin score and lung volumes, although no difference was observed in DLco No deaths occurred in either group over 24 months of follow up [44]. Based on these results, EULAR recommendations state that HSCT is a treatment option, but should only be considered in highly selected patients with rapidly progressive disease who are at risk of organ failure and given the high risk of treatment-related adverse effects and early mortality, the experience of the medical team is of high importance [33]. More recently, the SCOT trial in patients with diffuse cutaneous SSc and renal or pulmonary involvement demonstrated greater event-free survival with HSCT than CYC at 54 months (79% versus 50%) and at 72 months (74% versus 47%) [45].

#### Lung transplant

Carefully selected patients with SSc-ILD, who have not responded to treatment and who have no extrapulmonary contraindications, should be considered for lung transplant [46]. In a retrospective analysis of 30 patients with SSc-ILD who underwent lung or heart-lung transplant between 1993 and 2016, survival rates after 1, 3, and 5 years were 93, 76, and 60% [47]. Similarly, in a retrospective analysis of survival after lung transplant at a single US centre, survival in patients with SSc was 81% at 1 year and 66% at 5 years, similar to the rates observed in patients with other fibrotic ILDs [48]. However, lung transplantation in patients with SSc is possible in only a minority of patients, with transplant contraindicated in many cases due to active systemic disease, severe parietal thoracic involvement, and/or an increased risk of aspiration arising from oesophageal dysmotility and gastroparesis.

#### Supportive care

Patients with SSc-ILD should receive appropriate supportive care, which may include pulmonary rehabilitation, patient and caregiver education and other activities that aim to reduce symptoms and improve HRQL. Pulmonary rehabilitation has been shown to improve exercise capacity (6-min walk distance), dyspnoea and HRQL in patients with ILD [49]. However, further research is needed to establish the effectiveness of non-pharmacological interventions [50]. Palliative care should be available to patients at all stages of illness and should be individualised based on patient needs [51]. Management of comorbidities and treatment-related complications is an important part of the overall management of patients with SSc-ILD and should be part of routine care.

#### Investigational therapies

Based on the clinical and mechanistic similarities between SSc-ILD and idiopathic pulmonary fibrosis (IPF), the two approved therapies for IPF, nintedanib and pirfenidone, are being investigated as potential treatments for SSc-ILD. Nintedanib inhibits the proliferation, migration and differentiation of fibroblasts and the secretion of extracellular matrix, and has demonstrated antifibrotic, anti-inflammatory and vascular remodelling effects in animal models of SSc and ILD [52–54]. The efficacy and safety of nintedanib as a treatment for SSc-ILD are being assessed in the randomised placebo-controlled SENSCIS® trial (ClinicalTrials.gov NCT02597933; EudraCT 2015–000392-28) [55]. At baseline, participants were aged ≥18 years with first non-Raynaud symptom ≤7 years before screening, ≥10% fibrosis on HRCT of the lungs, FVC ≥40% predicted and DLco 30–89% predicted. Patients receiving low-dose prednisone and/or stable background therapy with MMF or methotrexate were eligible to participate. A total of 580 patients were randomised 1:1 to receive nintedanib 150 mg twice daily (bid) or placebo, stratified by the presence of anti-Scl-70/anti-topoisomerase I antibody. The primary endpoint is the annual rate of decline in FVC (mL/year) assessed over 52 weeks. Key secondary endpoints are absolute changes from baseline to week 52 in the mRSS and in the St George’s Respiratory Questionnaire (SGRQ) total score, which has been endorsed by an expert working group as a measure of HRQL in trials in SSc-ILD [56].

The precise mechanism of action of pirfenidone is unclear, but it has exhibited a number of effects in vitro and in animal models that may be relevant to its ability to slow the progression of pulmonary fibrosis, e.g.*,* inhibited proliferation and differentiation of fibroblasts and reduced synthesis of collagen [57–59]. This may be mediated, at least in part, through inhibitory effects on GLI transcription factors [60]. In the Phase II LOTUSS study, a 16-week open-label trial of pirfenidone in patients with SSc-ILD, the adverse event profile of pirfenidone was acceptable, similar to that seen in patients with IPF, and not affected by concomitant use of MMF, although a longer titration period may be associated with better tolerability [61]. The effects of pirfenidone vs placebo in patients with SSc-ILD who are receiving MMF are being investigated in SLS III (ClinicalTrials.gov NCT03221257). The RELIEF trial, which investigated the efficacy and safety of pirfenidone vs placebo given on top of anti-inflammatory therapy, in patients with progressive ILD of various etiologies [62] was terminated early due to slow recruitment and is yet to report results.

Interleukin-6 (IL-6), a pro-inflammatory cytokine, may play several roles in the pathogenesis of SSc, including promoting the differentiation of B-cells to immunoglobulin-secreting plasma cells, the differentiation of T-cells towards a Th17 phenotype, and the transformation of fibroblasts to myofibroblasts [63]. The efficacy and safety of an anti-IL-6 monoclonal antibody, tocilizumab, in patients with SSc have been investigated in two trials. In the Phase II faSScinate trial in patients with progressive SSc (*N* = 87), there was no significant difference between tocilizumab and placebo in the primary endpoint of change in mRSS at week 24 but exploratory analyses suggested that tocilizumab may be associated with clinically relevant improvements in lung function [64]. In the Phase III focuSSced trial in 210 patients with SSc, the primary endpoint of change in mRSS at week 48 was not met. In exploratory analyses, the mean change from baseline in FVC at week 48 was − 0.4% predicted in the tocilizumab group versus − 4.6% predicted in the placebo group and the proportion of patients with a decline in FVC of > 10% predicted at week 48 was 5.4% with tocilizumab and 16.5% with placebo [65].

Several other compounds, with a variety of mechanisms of action, are being investigated as potential therapies for SSc-ILD in Phase II/III clinical trials [Table 1].

**Table 1:** Ongoing and recently completed Phase II/III randomized controlled trials of potential treatments for SSc-ILD listed on ClinicalTrials.gov

Agent (company)	Type of molecule	Trial name (ClinicalTrial.gov identifier)	Population; sample size	Lung function endpoint/s	Estimated primary completion date^a^
Lanifibranor (Inventiva Pharma)	Peroxisome proliferator-activated receptor agonist	FASST (NCT02503644)	Patients with dcSSc; *n* = 132	Changes from baseline in FVC % predicted and DLco % predicted at weeks 24 and 48 (secondary endpoints)	October 2017
Anabasum / lenabasum (Corbus Pharmaceuticals)		RESOLVE-1 (NCT03398837)	Patients with dcSSc; *n* = 354	Change from baseline in FVC at week 53 (secondary endpoint)	March 2020
Riociguat (Bayer)	Guanylate cyclase stimulator	RISE-SSc (NCT02283762)	Patients with dcSSc; *n* = 121	Change from baseline in FVC % predicted at week 52 (secondary endpoint)	October 2018 (actual)
Tocilizumab (Hoffmann-La Roche)	Interleukin-6 receptor antagonist	focuSSced (NCT02453256)	Patients with SSc and mRSS of 10–35; *n* = 212	Change from baseline in FVC at week 48 (secondary endpoint)	January 2018 (actual)
Abatacept (Bristol-Myers Squibb)	Elective T-cell costimulation modulator	ASSET (NCT02161406)	Patients with dcSSc; *n* = 88	Change from baseline in FVC % predicted at week 52 (secondary endpoint)	September 2018
Nintedanib (Boehringer Ingelheim)	Tyrosine kinase inhibitor	SENSCIS (NCT02597933)	Patients with SSc-ILD; *n* = 580	Annual rate of decline in FVC (mL/year) over 52 weeks (primary endpoint)	October 2018
Ifetroban (Cumberland Pharmaceuticals)	Antagonist of thromboxane A2 / prostaglandin endoperoxide receptor	NCT02682511	Patients with dcSSc (*n* = 14) or SSc-PAH (*n* = 20)	Changes from baseline in FVC and DLco at weeks 12, 26, 52 (secondary endpoints)	December 2019
Pirfenidone (Genentech)	Pyridone analogue	NCT03068234	Patients with SSc; *n* = 72	Secondary endpoints: • Changes from baseline in FVC and DLco at weeks 24 and 52 • Assessment of chest CT at weeks 24 and 52	April 2019
Pirfenidone (Genentech)	Pyridone analogue	SLS III (NCT03221257)	Patients with SSc-ILD on background MMF; *n* = 150	Changes from baseline at month 18 in: • FVC % predicted (primary endpoint) • DLco % predicted • Mahler Modified Transitional Dyspnoea Index • Total lung capacity (HRCT) • SGRQ total score	April 2021
Rituximab (study funded by UK Medical Research Council and National Institute for Health Research)	CD20-directed cytolytic antibody	RECITAL (NCT01862926)	Patients with CTD-ILD; *n* = 116	Changes from baseline in FVC at week 24 (primary endpoint) and week 48 (secondary endpoint)	November 2019

^a^According to ClinicalTrials.gov (accessed 12 November 2018)

*CRISS* Combined Response Index in Diffuse Systemic Sclerosis, *CTD-ILD* connective tissue disease-associated interstitial lung disease;

## Conclusions

ILD is a common manifestation of SSc that is associated with early mortality. After diagnosis, all patients with SSc can benefit from an HRCT scan of the chest, PFTs, and assessment of respiratory symptoms. As the development or progression of ILD can occur at any time, patients should be monitored regularly, particularly in the first few years after diagnosis. Treatment should be considered when the disease is clinically significant, particularly when there is evidence of progression based on a decline in lung function, progression of fibrosis on HRCT, or worsening of respiratory symptoms. Currently treatment of SSc-ILD focuses on immunosuppressant therapies, particularly CYC and MMF. A number of new therapies with differing mechanisms of action are under active investigation.

## Abbreviations

ACR: American College of Rheumatology
bid: twice daily
CYC: Cyclophosphamide
dcSSc: diffuse cutaneous systemic sclerosis
DLco: Diffusion capacity of the lung for carbon monoxide
ERS: European Respiratory Society
EULAR: European League Against Rheumatism Collaborative Initiative
EUSTAR: EULAR Scleroderma Trials and Research Group
FVC: Forced vital capacity
HRCT: High resolution computed tomography
HRQL: Health-related quality of life
HSCT: Haematopoietic stem cell transplantation
ILD: Interstitial lung disease
IPF: Idiopathic pulmonary fibrosis
lcSSc: limited cutaneous systemic sclerosis
MMF: Mycophenolate mofetil
mRSS: modified Rodnan skin score
NSIP: Non-specific interstitial pneumonia
PFTs: Pulmonary function tests
PH, SGRQ: St George’s Respiratory Questionnaire
SLS: Scleroderma Lung Study
SSc: Systemic sclerosis
SSc-ILD: Interstitial lung disease associated with systemic sclerosis
